# KIFC1 Overexpression Promotes Pancreatic Carcinoma Progression via Stabilising BUB1B

**DOI:** 10.1111/jcmm.70767

**Published:** 2025-08-26

**Authors:** Ao Cui, Ying‐Xue Yu, Mei‐Xue Xiong, Ji‐Yang Wang, Ye‐Qing Zou, Ya‐Qiong Zhu, Long‐Jian Ran, Yu Zhang, Rui‐Xiang Liu, Ming‐Yi Dong, Hui Wang, Lu Fang, Xiao‐Wei Fu

**Affiliations:** ^1^ Department of General Surgery The Second Affiliated Hospital of Nanchang University Nanchang China; ^2^ Jiangxi Province Key Laboratory of Molecular Medicine Nanchang China; ^3^ Department of Neurology The Second Affiliated Hospital of Nanchang University Nanchang China; ^4^ Department of General Surgery The People's Hospital of Gongqingcheng Gongqingcheng China; ^5^ Department of Otorhinolaryngology The Second Affiliated Hospital of Nanchang University Nanchang China

**Keywords:** BUB1B, KIFC1, pancreatic cancer, protein interactions, tumorigenesis, ubiquitination

## Abstract

Pancreatic cancer (PC) is a highly lethal tumour of the gastrointestinal tract. New molecular targets are urgently needed for its treatment. Kinesin family member C1 (KIFC1) is implicated in the development and progression of several types of cancer. Previous studies from our group demonstrated that KIFC1 overexpression in hepatocellular carcinoma promotes malignant behaviours via the PI3K/AKT pathway. However, the molecular and functional mechanisms of KIFC1 in PC are not yet fully elaborated. In this study, KIFC1 and BUB1B were significantly upregulated in PC patient samples, and high KIFC1 expression was closely associated with the malignant phenotype and poorer overall survival (OS) in PC patients. Functional experiments showed that KIFC1 knockdown inhibited PC cell growth in vivo and in vitro, blocked cell cycle progression and hindered cell migration and invasion. In addition, rescue experiments showed that KIFC1 induced PC cell malignant behaviours dependent on BUB1B. Mechanistically, KIFC1 regulates BUB1B expression by competitively binding to BUB1B and reducing its ubiquitination and degradation. We have shown for the first time the molecular regulatory mechanism between KIFC1 and BUB1B on PC. Therefore, KIFC1 shows promise as an attractive therapeutic target for PC in the future.

**Abbreviations**
EMT: epithelial–mesenchymal transition
GEO: gene expression omnibus
IHC: immunohistochemistry
KIFC1: kinesin family member C1
OS: overall Survival
PC: pancreatic cancer
TCGA: The Cancer Genome Atlas Program

## 1 Introduction

Pancreatic cancer (PC) causes approximately 50,000 deaths each year in America. Unfortunately, most PC patients have a poor prognosis. Investigations have shown that the 5‐year survival rate for patients with PC is only 13% [1]. Surgical removal of the lesion is currently the only viable option for eradicating PC, but early metastasis or extensive local invasion renders resection for PC patients less than 20% [2, 3]. Although adjuvant and systemic chemotherapy are currently indispensable therapeutic methods for improving long‐term outcomes, their effects are still unclear [4].

In 1985, Ronald D. Vale discovered kinesins in squid giant axons that induce microtubule movement [5]. To date, 14 distinct families of kinesin superfamily proteins (KIFs) have been identified, ranging from KIF1 to KIF14 [6]. KIFs participate in a range of cellular processes, including synaptic vesicle transport, centrosome clustering and chromosomal transport during mitosis or meiosis, due to their function as molecular motors [7, 8]. Kinesin family member C1 (KIFC1) is a member of the kinesin‐14 family of C‐type kinesins. Previous research has shown that KIFC1 is linked to the development and progression of several types of tumours, including prostate carcinoma [9], breast carcinoma [10], gastric carcinoma [11], endometrial carcinoma [12] and non‐small cell lung carcinoma [13]. Our previous experiments showed that KIFC1 is significantly overexpressed in hepatocellular carcinoma and contributes to the progression of the disease, resulting in a poor prognosis [14]. However, subsequent studies by Zhang et al. utilised functional tests to establish a correlation between KIFC1 and PC progression [15], while Deng et al. proceeded to investigate the regulatory function of the DD6X/KIFC1 axis in PC progression [16]. However, our research has revealed more interesting and specific molecular mechanisms of KIFC1 in PC progression.

The BUB1B protein plays a crucial regulatory role in mitosis, as demonstrated by recent studies [17]. It encodes a protein kinase that activates the spindle checkpoint by phosphorylating members of the mitotic checkpoint complex [18, 19, 20]. Various types of cancer, including bladder [21], pancreatic [22] and breast cancers [23], have been linked to mutations in BUB1B. According to Xin Fu et al., BUB1B was significantly enriched in the WNT signalling pathway [24]. FBXW7 has E3 ubiquitination ligase activity, and Vishnu M. Nair et al. reported that BUB1B can undergo ubiquitination degradation by FBXW7 [25]. However, the function and molecular mechanism of BUB1B in PC have not been reported.

The Wnt signalling pathway was initially identified in a mouse mammary tumour virus model and was designated Int‐1 by Roel Nusse [26]. The Wnt/β‐catenin signalling pathway is a canonical signalling pathway in which the β‐catenin protein plays a key role. In the nucleus, β‐catenin binds to DNA‐binding proteins of the T‐cell factor/lymphoid enhancing factor (TCF/LEF) family, leading to the activation of downstream target genes. Wnt pathway activation is inhibited by the degradation of β‐catenin, which is phosphorylated by a disruption complex composed of APC, Axin and GSK3β. This phosphorylation recruits the E3 ubiquitin ligase containing β‐TrCP to degrade β‐catenin [27].

However, the molecular interactions between the KIFC1, BUB1B and Wnt signalling pathways in PC have not been fully elucidated. Our study revealed that KIFC1 stabilises BUB1B, ultimately activating the Wnt pathway and promoting PC development. This study provides a new molecular mechanism of KIFC1 in PC development and identifies new targets for the future treatment of PC.

## 2 Materials and Methods

### 2.1 Patients and Tissue Samples

This study included 62 patients with PC who were pathologically diagnosed between 2016 and 2023 and who underwent pancreatectomy at the Department of Hepatobiliary Surgery of the Second Affiliated Hospital of Nanchang University. The specimens were subjected to immunohistochemical analysis after being embedded in formalin and paraffin. The patients' clinicopathological data were also collected. All patients provided informed consent. Tumour staging was determined according to the Union for International Cancer Control TNM classification guide (8th edition, 2019). The Ethics Committee of the Second Affiliated Hospital approved the study (Approval No. Review [2021] No. 039).

### 2.2 Cell Lines

PANC‐1, SW‐1990, BXPC‐3, ASPC‐1 and H6C7 (human normal pancreatic ductal epithelial cells) cells were purchased from the Institutes for Life Sciences, Chinese Academy of Sciences (Beijing, China). SW1990, H6C7 and other cell lines were cultured in DMEM (Thermo Fisher, 12,430,054, USA) supplemented with 10% foetal bovine serum (FBS, HyClone, SH30396.02, USA) and 1% penicillin–streptomycin, in a humidified incubator at 37°C with 5% CO_2_. ASPC‐1 cells were maintained in RPMI‐1640 medium (Thermo Fisher, 11,875,119, USA) with 10% FBS under the same incubation conditions. The culture medium was refreshed every 2–3 days.

### 2.3 Immunohistochemistry

Tissue specimens were dewaxed in xylene and rehydrated in a graded series of ethanol. Afterwards, the tissue sections were placed in a pressure cooker at 100°C for 15 min to repair the antigens. Subsequently, the sections were incubated with H_2_O_2_ for 15 min at room temperature to block endogenous peroxidase activity. Next, the sections were blocked with goat serum (Thermo Fisher, 16,210,064, USA) for 30 min. Next, the sections were incubated with an anti‐KIFC1 antibody (1:200, ORIGENE, TA38608) at 4°C overnight. Afterwards, the sections were incubated with the corresponding secondary antibodies. Both the staining intensity and area of positive staining for KIFC1 were evaluated by two pathologists in a mutually blinded manner. Staining was graded as 0 (negative), 1 (weakly positive), 2 (moderate) or 3 (strongly positive) based on the intensity of staining. The extent of staining was scored as 1 (< 10%), 2 (10%–40%), 3 (40%–75%) or 4 (>75%). The intensity and extent of the staining were multiplied to obtain a total staining score. The KIFC1 score ranged from 0 to 12, which allowed the specimens to be categorised into a low‐expression group (0–3) and a high‐expression group [3–12].

### 2.4 Data Mining and Bioinformatics Analysis

The expression profiles of KIFC1 and BUB1B in PC and their respective survival curves were obtained from the Gene Expression Profiling Interactive Analysis (GEPIA) online database (http://gepia.cancer‐pku.cn/). KIFC1 and BUB1B gene correlation analyses were performed with the Gene_Corr module of the TIMER2.0 database (http://timer.cistrome.org/). The PPIs were first identified from the STRING database and then mapped using Cytoscape v3.9.0 software. Hub genes were identified using the CytoHubba plugin in Cytoscape based on the Degree algorithm. The node with the highest degree was highlighted as the central hub and visually represented by a larger size and deeper red colour, indicating greater connectivity. The GSE107160, GSE16515, and GSE15471 datasets were obtained from the GEO database (https://www.ncbi.nlm.nih.gov/geo/).

### 2.5 Cell Transfection and Reagents

Small interfering RNA (siRNA) targeting KIFC1 and lentiviruses for the knockdown or overexpression of KIFC1 and BUB1B were purchased from General Biol (Anhui, China). The recombinant plasmids used were obtained from Gene Pharma (Shanghai, China). Cells were transfected using Lipofectamine 2000 (Invitrogen, 11,668–019, USA) according to the manufacturer's protocol to obtain stable overexpression or knockdown cell lines. AZ82 (AZ82, a KIFC1 inhibitor) was procured from Cayman (Michigan, USA) for the purpose of inhibiting kinesin. The sequences of the siRNAs used were as follows: siRNA1: 5′‐GGACUUAAAGGGUCAGUUATT‐3′ and siRNA2: 5′‐CGGGAACGCCUUCGGGAAATT‐3′.

### 2.6 Quantitative Real‐Time PCR (qRT–PCR)

The expression of KIFC1 was assessed by qRT–PCR. Total RNA was isolated from PC cells using TRIzol reagent, and cDNA was obtained by reverse transcription according to the instructions of TRAN One‐Step gDNA Removal and cDNA Synthesis SuperMix Kits (TransGen, AE311, China). qRT–PCR was subsequently performed using SYBR Premix Ex Taq II Kits (Takara, RR420B, USA). The 2^−ΔΔCt^ method was used to calculate KIFC1 using GAPDH as an internal reference [28] for relative expression. The sequences of the primers used were as follows: KIFC1‐F: 5′‐GCAGGAACTCAAGGGCAA‐3′, *KIFC1*‐R: 5′‐GCTAAGGCGGGGTTGGAG‐3′; and GAPDH‐F: 5′‐GGACCTGACCTGCCGTCTAG‐3′; GAPDH‐R: 5′‐GTAGCCCAGGATGCCCTTGA‐3′.

### 2.7 Western Blotting and In Vitro Ubiquitination Assay

Cell or tissue samples were lysed using RIPA lysis buffer supplemented with protease inhibitors. The proteins were separated by SDS–PAGE using either an 8% or 10% gel and then transferred to 0.22 μm PVDF membranes (Millipore, ISEQ00010, USA). The membranes were blocked with 5% skim milk and incubated with primary antibodies overnight at 4°C. The strips were then washed with TBST and incubated with secondary antibodies for 1 h at room temperature. Finally, the strips were visualised using a bioimaging system and analysed with ImageJ software. For the in vitro ubiquitination assay, PC cells subjected to KIFC1 knockdown or overexpression were exposed to MG132 treatment for 6 h before harvesting. The cell lysates were prepared and immunoprecipitated with anti‐BUB1B antibody. The ubiquitination level of BUB1B was assessed using an anti‐Ub antibody.

Primary antibodies against Ub (Abcam, ab134953), KIFC1 (cat. no. TA384608, ORIGENE), BUB1B (cat. no. HA60053, HUABIO), β‐catenin (cat. no. #8480, Cell Signaling Technology), TCF‐4 (cat. no. #2569, Cell Signaling Technology), c‐Myc (cat. no. #9402, Cell Signaling Technology), cyclin D1 (cat. no. #55506, Cell Signaling Technology), N‐cadherin (cat. no. #13116, Cell Signaling Technology), E‐cadherin (cat. no. #3195, Cell Signaling Technology), vimentin (cat. no. #5741, Cell Signaling Technology) and GAPDH (cat. no. #5174, Cell Signaling Technology) were used.

### 2.8 Cell Counting Kit‐8

The transfected cells were inoculated into 96‐well plates at a density of 4000 cells per well and cultured for 24, 48 or 72 h. Then, 10 μL of CCK‐8 (Abcam, ab228554, USA) reagent was added to each well at the end of each incubation cycle. After a 2 h incubation, the absorbance of each well at 450 nm was measured using a microplate reader.

### 2.9 Colony Formation Assay

Cells that had been transfected and resuspended were inoculated into six‐well plates at a density of 800 cells per well. The cells were cultured for 14 days, and the cells in each well were fixed with paraformaldehyde for 30 min at room temperature and then stained with crystal violet for 10 min. A microscope was used to count the number of cell clusters.

### 2.10 Cell Proliferation Assay

Cell proliferation was assessed by a 5‐ethynyl‐2'deoxyuridine (EdU) proliferation assay, and an EdU kit (UElandy, C6044S, China) was purchased from UElandy Biotechnology Co. in Suzhou, China. Transfected and resuspended cells were inoculated into 96‐well plates at 1 × 10^5^ cells per well. Once the cells had attached to the wall, the EdU reagent was added to the medium, and the cells were incubated for 2 h. After this, the cells were fixed with 4% paraformaldehyde and destained with glycine, and the cell membrane was permeabilised with 0.5% Triton X‐100. YF 594 or YF 488 was added for 30 min at 25°C in the dark. After washing, Hoechst 33,342 was added for another 30 min under the same conditions. Images were acquired using a fluorescence microscope.

### 2.11 Wound Healing

The fused cells were treated with mitomycin (1 μg/mL) for 1 h after being switched to serum‐free medium. Vertical scratches were made at the bottom using a 200‐μL pipette gun tip. The shed cells were washed away with PBS, and the scratches were imaged at 0 h. Images were captured again 24 h after incubation, and the rate of cell migration was calculated.

### 2.12 Transwell Assay

For the invasion assay, Matrigel was spread into Transwell chambers. Transfected cells were then resuspended in serum‐free DMEM and added to the upper chambers, while DMEM containing 10% FBS was added to the lower chambers. The chambers were incubated for 48 h in a 24‐well plate. After incubation, the chambers were fixed with 4% paraformaldehyde for 25 min and stained with crystal violet for 5 min. Images were captured using a microscope, and the cells were counted.

### 2.13 Cell Cycle Assay

A total of 1 × 10^5^ posttransfection cells were collected and fixed with precooled 70% ethanol in a refrigerator at 4°C overnight. The following day, the cells were washed twice with PBS, stained with 0.5 mL of PI/RNase (Abcam, ab112116, USA) and resuspended. The cells were incubated for 30 min at room temperature in the dark. Afterwards, the cell cycle distribution was analysed using flow cytometry.

### 2.14 Animal Experiments

Female BALB/c nude mice (6–8 weeks old) weighing 15 ± 1 g from Beijing SPF Biotechnology Co. Ltd. were used to construct a xenograft tumour model. All animal experiments were conducted at Nanchang Royo Biotech Co. Ltd. The aim of this study was to investigate the effect of KIFC1 on tumour formation in vivo.

The mice were injected subcutaneously with 1 × 10^6^ PANC‐1 cells (with stable KIFC1 knockdown) resuspended in 100 μL of serum‐free DMEM. Tumour volumes were measured at 5‐day intervals, and images of the mice were captured after 30 days using a small animal live imager. After the mice were euthanized, the tumour tissue was removed for detection. All animal experiments were approved by the Institutional Animal Care and Use Committee of Nanchang Royo Biotech Co. Ltd. (Nanchang, China, IACUC No. RYE2024033001).

### 2.15 Statistical Analysis

All analyses were performed using SPSS 26.0 software. Survival curves were plotted using the Kaplan–Meier method and compared using the log‐rank test, and the association between KIFC1 and clinicopathology was tested using the χ ^2^ test. Bivariate correlations were calculated using Pearson's correlation coefficient. Comparisons between two groups were made using two independent samples *t* tests, and differences were considered statistically significant at *p* < 0.05. The data are presented as mean ± SD, and *p* < 0.05 was considered to indicate statistical significance.

## 3 Results

### 3.1 KIFC1 Is Highly Expressed in PC Tissue and IS Associated With Advanced Clinical Stage and Poor Prognosis

**FIGURE 1:**
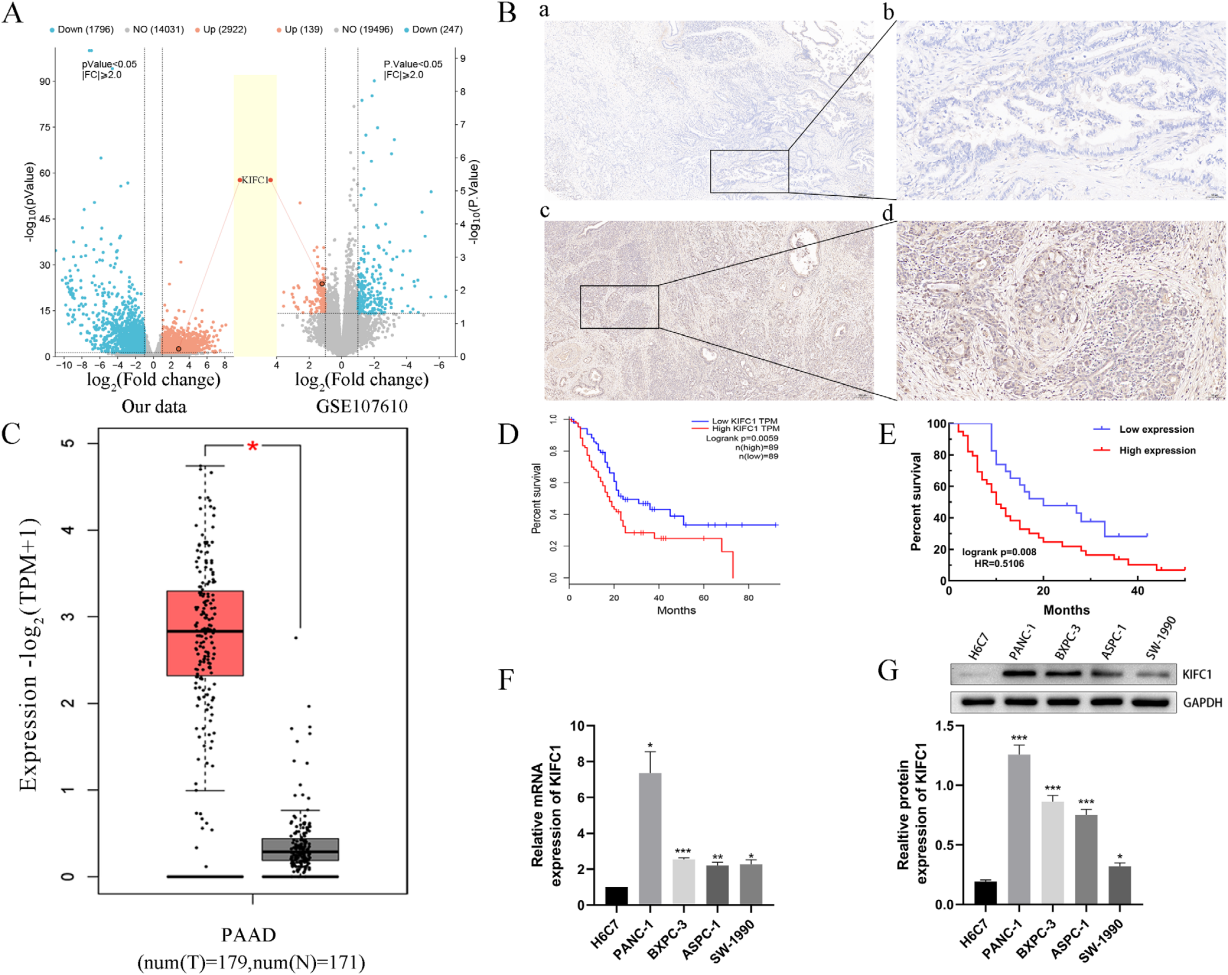
KIFC1 is expressed at high levels in both pancreatic cancer tissues and cells. (A) The volcano plot was constructed using the fold change values and *p* values. Red dots represent upregulated genes blue dots represent downregulated genes, and grey dots represent genes whose expression did not significantly change. KIFC1 was jointly screened using our high‐throughput sequencing data and the GSE107160 dataset. (B) Immunohistochemical (IHC) staining of clinical human pancreatic adenocarcinoma and corresponding paracancerous tissue samples: (a) × 40 normal pancreatic tissue sample, (b) × 400 normal pancreatic tissue sample, (c) × 40 pancreatic tumour tissue sample, on in PC tissues (*n* = 179) compared to normal pancreatic tissues (*n* = 171). (D, E) Overall survival (OS) of 62 PC patients from our collected data and OS of 178 PC patients from the GEPIA database. (F, G) RT–qPCR and western blotting were used to measure the expression of KIFC1 mRNA and protein in H6C7, PANC‐1, BXPC‐3, ASPC‐1 and SW‐1990 cells. Values are presented as mean ± SD. *p < 0.05, **p < 0.01, ***p < 0.001.

Four pancreatic adenocarcinoma and paracarcinoma tissue samples were utilised for the purpose of sequencing. The intersection of these samples with the GSE107160 dataset subsequently revealed elevated levels of KIFC1 expression (Figure 1A). To explore the expression and location of KIFC1 in PC patients, we performed immunohistochemistry (IHC) analysis on 62 PC patient samples. We observed almost no KIFC1 expression in normal tissues. On the other hand, KIFC1 expression was significantly high in neoplastic tissues, and it was detected mainly in the nuclei of tumour cells (Figure 1B). This finding was supported by findings obtained from the online GEPIA database (Figure 1C). Furthermore, by analysing the clinicopathological features of 62 patients, we found that a high KIFC1 expression level was closely correlated with T stage (*p* = 0.016), TNM stage (*p* = 0.017) and histological grade (*p* = 0.001) in patients with PC (Table 1). These findings indicate that KIFC1 expression is clearly related to poor clinical and pathological stage. Therefore, we analysed the association between KIFC1 expression and patient survival by Kaplan–Meier analysis, and the results demonstrated that KIFC1‐high PC patients had a significantly worse overall survival (OS) than did KIFC1‐low patients (log rank *p* = 0.008; Figure 1E). This result was also confirmed by the GEPIA database (log rank *p* = 0.0059; Figure 1D).

**TABLE 1:** Relationships between KIFC1 expression and the clinicopathological features of 62 PC patients.

Variables	*n*	KIFC1		*χ* ^2^	*p*
		High expression	Low expression		
Sex				0.466	0.495
Male	25	17 (68.0%)	8 (32.0%)		
Female	37	22 (59.5%)	15 (40.5%)		
Age (years)				0.726	0.394
≥ 60	34	23 (67.6%)	11 (32.4%)		
< 60	28	16 (57.1%)	12 (42.9%)		
T staging				8.290	0.016*
T1	20	8 (40.0%)	12 (60.0%)		
T2	27	18 (66.7%)	9 (33.3%)		
T3	15	13 (86.7%)	2 (13.3%)		
Nerve infiltration				0.777	0.378
Yes	47	31 (66.0%)	16 (34.0%)		
No	15	8 (53.3%)	7 (46.6%)		
Venous invasion				2.503	0.114
Yes	35	25 (71.4%)	10 (28.6%)		
No	27	14 (51.9%)	13 (48.1%)		
Histological grade				11.519	0.001*
High	19	6 (31.6%)	13 (68.4%)		
Low/Medium	43	33 (76.7%)	10 (23.3%)		
Lymph node status				0.521	0.470
Negative	26	15 (57.7%)	11 (42.3%)		
Positive	36	24 (66.7%)	12 (33.3%)		
TNM staging				8.127	0.017*
I	19	7 (36.8%)	12 (63.2%)		
II	29	21 (72.4%)	8 (27.6%)		
III	14	11 (78.6%)	3 (21.4%)		

### 3.2 KIFC1 Overexpression Promotes PC Cell Growth In Vitro

**FIGURE 2:**
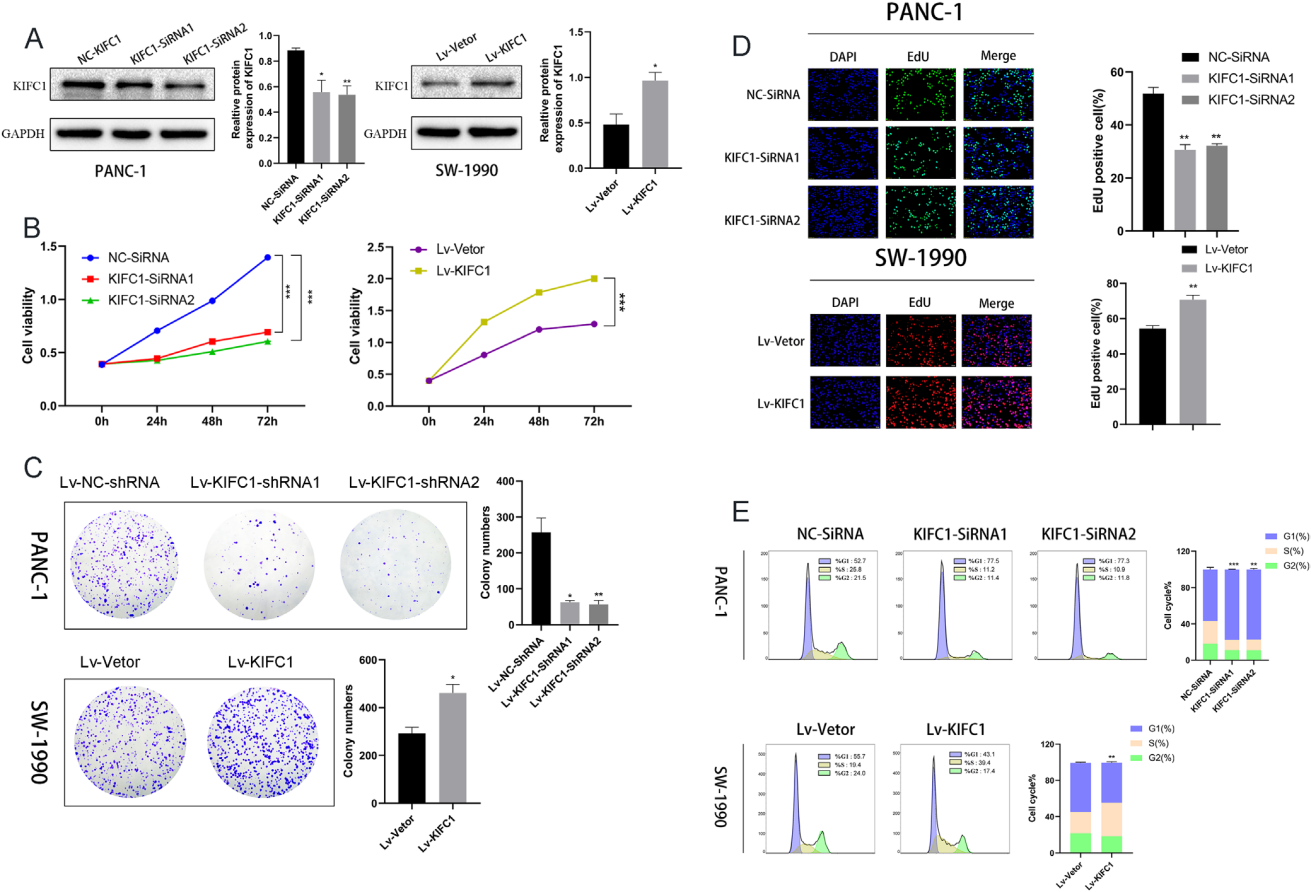
KIFC1 promotes the proliferation and cell cycle progression of PC cells. (A) Western blotting demonstrated that SiRNAs reduced KIFC1 expression, while overexpression of KIFC1 lentivirus increased KIFC1 expression. (B) CCK‐8 assays showed that siRNA‐KIFC1 inhibited PC cell viability, while KIFC1 overexpression improved cell viability. (C) Colony formation assays revealed that shRNA‐KIFC1 reduced the colony‐forming ability of cells, while KIFC1 overexpression increased the colony‐forming ability of cells. (D) EdU assay revealed that the proliferative capacity of KIFC1‐knockdown cells decreased and that of KIFC1‐overexpressing cells was increased. (E) KIFC1 knockdown caused cell cycle arrest in the G1 phase, as determined by flow cytometry, while KIFC1 overexpression promoted cell cycle progression from the G1 phase to the S and G2 phases. Values are presented as mean ± SD. *p < 0.05, **p < 0.01, ***p < 0.001.

The KIFC1 protein and mRNA were examined by western blotting and qRT–PCR, and the results revealed that KIFC1 was more highly expressed in PC cell lines than in normal pancreatic ductal epithelial cells (Figure 1F,G). To confirm that KIFC1 promotes PC cell proliferation, two independent siRNAs and one KIFC1‐overexpressing lentiviral vector were used to silence or overexpress KIFC1, respectively. SiRNA transfection and lentivirus infection were detected in PANC‐1 and SW‐1990 cells via western blotting, and the KIFC1 protein levels decreased and increased, respectively (Figure 2A). Compared with control cells, SW‐1990 cells overexpressing KIFC1 exhibited greater cell viability, colony formation and proliferation (Figure 2B–D). However, KIFC1 knockdown in PANC‐1 cells had opposite effects on cell viability, colony formation and proliferation (Figure 2B–D). Furthermore, a cell cycle assay demonstrated that KIFC1 promoted progression through the cell cycle from the G1 to the S and G2 phases (Figure 2E).

### 3.3 KIFC1 Promoted PC Cell Migration and Invasion

**FIGURE 3:**
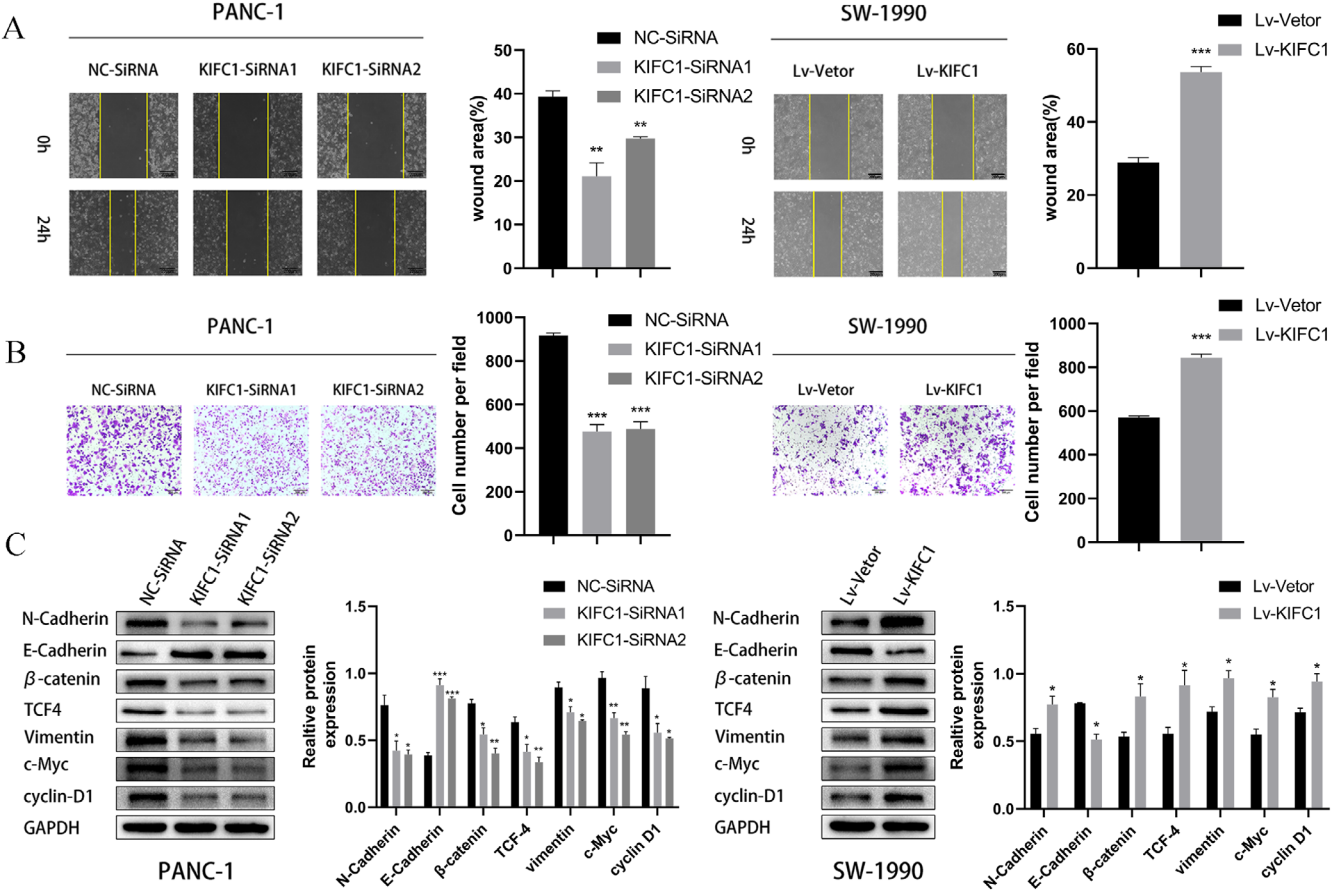
KIFC1 promotes PC cell migration, invasion, EMT and activation of the Wnt/β‐catenin pathway. (A) Scratch assay was used to detect the effect of KIFC1 on cell migration, and KIFC1 knockdown cells had decreased migration ability, while the opposite was true for the overexpression group. (B) Transwell assays showed that KIFC1 knockdown reduced cell invasion, while KIFC1 overexpression increased cell invasion. (C) Western blotting demonstrated that knockdown of KIFC1 reduced the protein expression of N‐cadherin, β‐catenin, TCF4, vimentin, C‐Myc and cyclin D1 and increased the protein expression of E‐cadherin, while overexpression of KIFC1 had the opposite effect. Values are presented as mean ± SD. *p < 0.05, **p < 0.01, ***p < 0.001.

Scratch and Transwell assays were performed in PANC‐1 and SW‐1990 cells to examine the impact of KIFC1 on PC cell migration and invasion. A scratch assay indicated that KIFC1 overexpression markedly enhanced migration, while the opposite effect was observed in the control group (Figure 3A). In addition, Transwell assays revealed that PC invasion capacity was closely related to KIFC1 expression (Figure 3B). It is widely known that epithelial–mesenchymal transition (EMT) is an indispensable factor that promotes cell migration and invasion [29, 30]. Therefore, western blotting was used to verify the key role of EMT in PC cell migration and invasion. The results demonstrated that the epithelial marker E‐cadherin was repressed; in contrast, the mesenchymal markers N‐cadherin and vimentin were increased in KIFC1‐overexpressing PC cells. However, silencing KIFC1 had the opposite effect (Figure 3C). Overall, the above results indicated that KIFC1 promoted PC cell migration and invasion.

### 3.4 KIFC1 Overexpression Activated the Wnt/β/Catenin Pathway in PC Cells

In a previous study, the Wnt/β/catenin pathway was verified to contribute to PC malignancy [31]. The dephosphorylation of β‐catenin prevents it from being degraded by ubiquitination, after which it accumulates in the nucleus and increases the expression of TCF/LEF. TCF/LEF subsequently interacts with downstream targets, such as c‐Myc and cyclin D1 [32]. Western blotting revealed that KIFC1 overexpression upregulated β‐catenin, TCF4, c‐My, and cyclin D1, while KIFC1 depletion downregulated the expression of these genes (Figure 3C). Thus, the results indicated that KIFC1 might affect PC malignancy by activating the Wnt/β‐catenin signalling pathway.

### 3.5 BUB1B Interacts With KIFC1 at the Protein Level, Is Highly Expressed in PC Patients and Is Associated With Poor Prognosis

**FIGURE 4:**
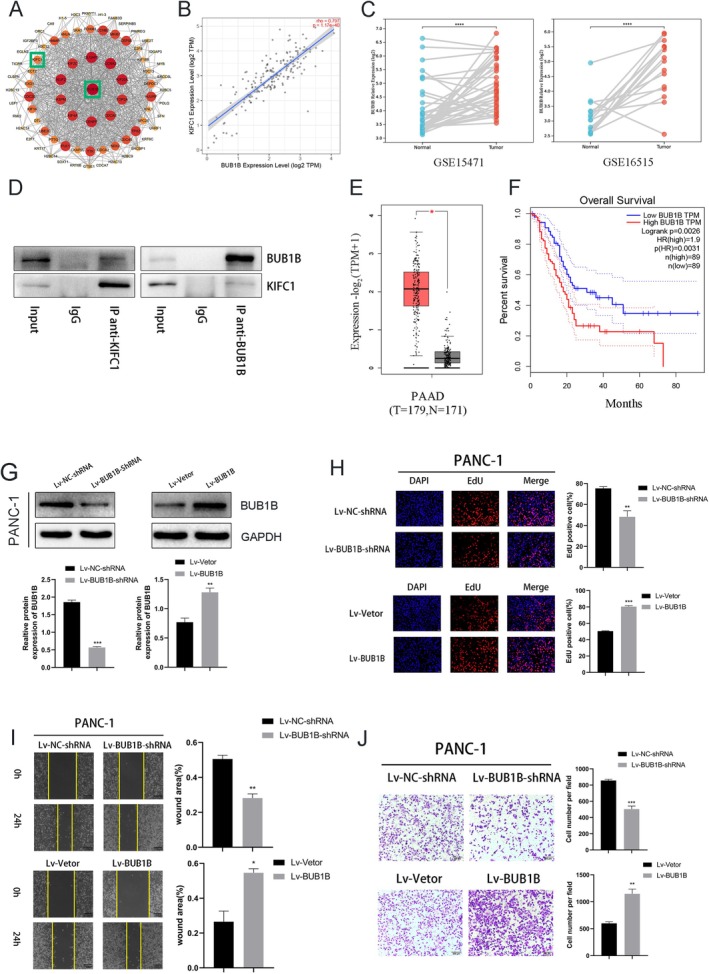
KIFC1 interacts with BUB1B and BUB1B affects PC cell proliferation, migration and invasion. (A) Upregulated hub genes identified by high‐throughput sequencing. The PPI network revealed that KIFC1 is correlated with BUB1B. (B) TIMER 2.0 database analysis showed that KIFC1 is strongly correlated with BUB1B expression. (C) Co‐IP experiments demonstrated that KIFC1 interacts with BUB1B at the protein level. (D) Both GSE15471 and GSE16515 indicate that BUB1B expression was greater in cancer tissues than in adjacent paracancerous tissues. (E) GEPIA database indicated that BUB1B expression is significantly greater in PC tissues than in normal pancreatic tissues. (F) In the GEPIA database, high expression of BUB1B was associated with a poor prognosis. (G) Western blotting confirmed that the BUB1B knockdown and overexpression lentiviruses effectively silenced and overexpressed BUB1B, respectively. (H—J) Positive effects of BUB1B on the proliferation, migration and invasion of PANC‐1 cells. Values are presented as mean ± SD. ****p < 0.0001.

These 2922 up‐regulated genes were selected from our sequencing data using the criteria of'*p* value < 0.05 and logFC > '. To identify key regulators, a PPI network was constructed from upregulated genes using STRING and visualised in Cytoscape. Degree‐based analysis via CytoHubba ranked BUB1B as the top hub gene, shown as the largest and deepest red node in the circular layout, indicating its central role in the network (Figure 4A). To investigate the relationship between KIFC1 and BUB1B in PC, we analysed transcriptomic data from The Cancer Genome Atlas (TCGA) Spearman's correlation analysis confirmed a strong positive correlation between the expression of KIFC1 and that of BUB1B in PC (*r* = 0.835; Figure 4B). Moreover, interactions between KIFC1 and BUB1B were observed at the protein level via Co‐IP assays (Figure 4C). To confirm the relationship between BUB1B expression levels and clinical outcomes, we selected two datasets (GSE15471 and GSE16151) from the GEO database for paired‐sample *t* tests. The results showed that BUB1B expression was significantly greater in tumour tissues than in adjacent normal tissues (Figure 4D). Additionally, the GEPIA database revealed high expression of BUB1B in patients with tumours, which adversely affected patient prognosis (Figure 4E,F).

### 3.6 BUB1B Mediates the Effect of KIFC1 on the Wnt/β‐Catenin Pathway and the Malignancy of PC Cells

**FIGURE 5:**
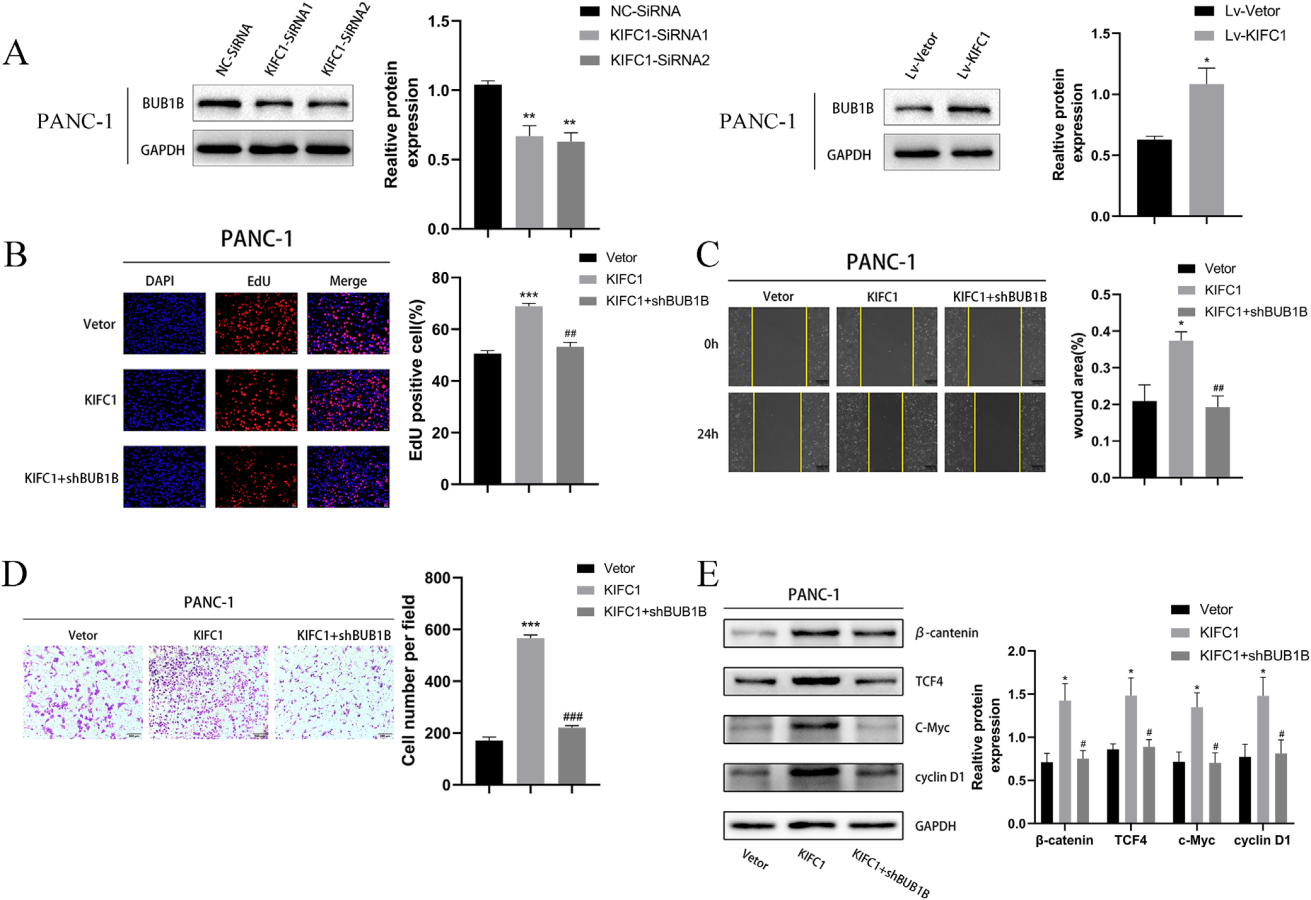
KIFC1 activates the WNT/β‐catenin pathway through BUB1B, resulting in the malignant behaviours of PC cells. (A) KIFC1 positively regulates the protein expression of BUB1B in PC cells. (B—E) Increase in PC cell proliferation, migration, invasion and activation of the Wnt/β‐catenin pathway induced by KIFC1 overexpression was reversed upon knockdown of BUB1B. Values are presented as mean ± SD. *p < 0.05, **p < 0.01, ***p < 0.001.

Western blotting revealed that BUB1B was successfully knocked down or overexpressed (Figure 4G). EdU experiments revealed that BUB1B knockdown decreased the proliferation of PC cells, whereas BUB1B overexpression had the opposite effect (Figure 4H). Similarly, a positive correlation between BUB1B expression and cell migration and invasion ability was observed in the scratch and Transwell assays (Figure 4I,J). The knockdown and overexpression of KIFC1 in PANC‐1 cells resulted in a similar trend for BUB1B (Figure 5A). A lentivirus was used to knock down BUB1B, which reversed the positive effects of KIFC1 overexpression on the proliferation, migration and invasion ability of PANC‐1 cells (Figure 5B–D). Similarly, decreased protein levels of β‐catenin, TCF‐4, c‐Myc and cyclin D1 were observed in KIFC1‐overexpressing cells upon BUB1B silencing (Figure 5E). These results demonstrated that reducing BUB1B expression inhibits the activation of the Wnt/β‐catenin pathway induced by KIFC1 overexpression.

### 3.7 KIFC1 Enhances PC Growth and Activation of the Wnt/β‐Catenin Pathway In Vivo

**FIGURE 6:**
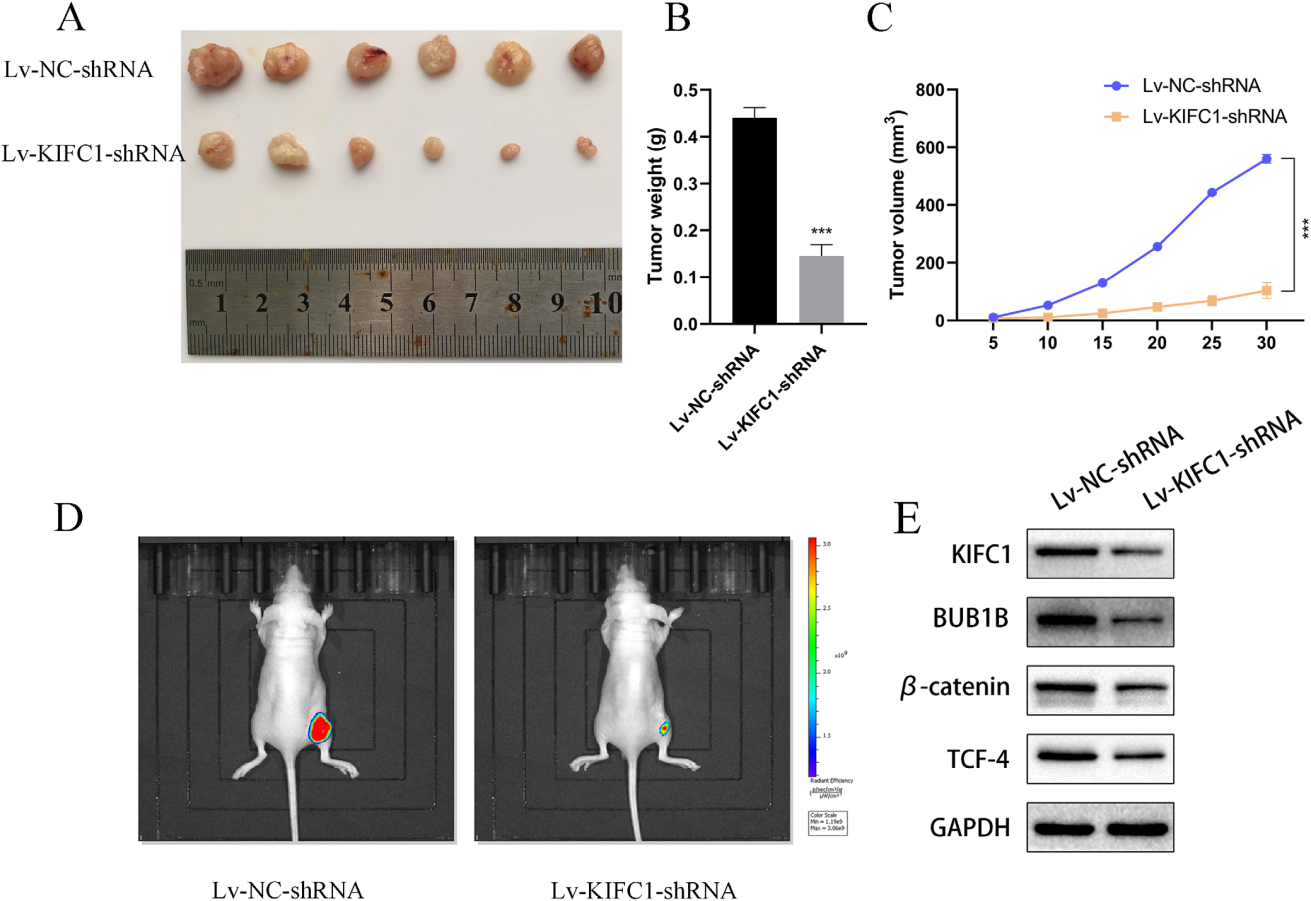
KIFC1 promotes PC proliferation and activation of the BUB1B/WNT/β‐catenin pathway in vivo. (A) Representative images of subcutaneous tumour nodule size in mice on day 30. (B, C) Final tumour mass bar graphs and line graphs of subcutaneous tumour growth versus time. (D) Representative images of subcutaneous tumours in mice on day 30 were acquired using an animal living imager. (E) Western blotting showed that KIFC1 regulates the downstream BUB1B and Wnt/β‐catenin pathways in vivo. Values are presented as mean ± SD. ***p < 0.001.

We established subcutaneous tumour models to explore the function of KIFC1 in regulating PC tumourigenesis. Tumours with silenced KIFC1 exhibited significantly lower growth rates, weights and volumes than those in the control group (Figure 6A–C). Moreover, a small animal imaging system was used to evaluate tumour growth, and the results also verified that KIFC1 knockdown obviously suppressed cancer cell proliferation (Figure 6D). Furthermore, Western blotting revealed that KIFC1 regulates BUB1B at the protein level and affects the activation of the Wnt/β‐catenin pathway in vivo (Figure 6E). Thus, in vivo experiments confirmed that KIFC1 regulates BUB1B and the downstream Wnt/β‐catenin pathway, promoting tumour growth.

### 3.8 KIFC1 and FBXW7 Competitively Bind BUB1B

**FIGURE 7:**
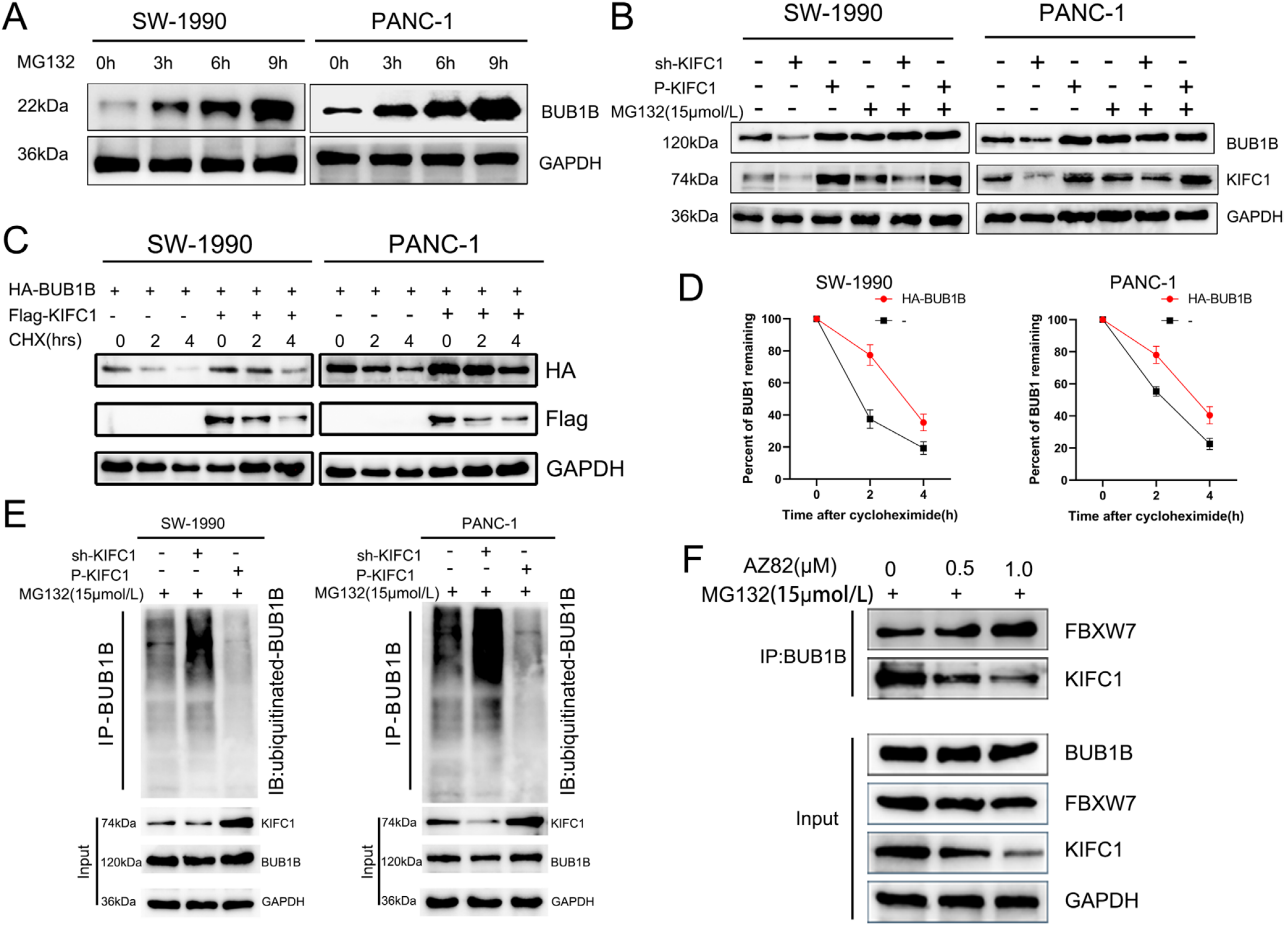
KIFC1 prevents BUB1B ubiquitination degradation by competitive binding to FBXW7. (A) Proteasome inhibitor MG132 was administered to PC cells, resulting in an increase in BUB1B expression levels over time. (B) Expression levels of BUB1B remained unaltered in PC cells exhibiting KIFC1 overexpression and knockdown following the addition of MG132. (C, D) Following the addition of cycloheximide for the specified durations of treatment, the degradation of HA‐BUB1B and the presence or absence of Flag‐KIFC1 were determined using anti‐HA and anti‐Flag antibodies. (E) PC cell lysates transduced with sh‐KIFC1 and P‐KIFC1 plasmids were treated with MG132 for 4 h. Following this, the lysates were collected for immunoprecipitation using anti‐ubiquitin (Ub) antibody, and then subjected to immunoblotting using YAP1 antibody. (F) PC cells were treated with varying doses of the KIFC1 inhibitor AZ82 for a period of 4 h. Following this, lysates were collected for subsequent immunoprecipitation and immunoblotting.

To gain further insight into the precise mechanism by which KIFC1 stabilises BUB1B, we explored the evidence that BUB1B has been shown to be degraded via the ubiquitination pathway [25]. It can be postulated that KIFC1 may competitively bind BUB1B with FBXW7, thereby preventing the ubiquitination of BUB1B by FBXW7. To test this hypothesis, the cells were treated with MG132(15 μM) for 0, 3, 6 and 9 h, respectively. The results demonstrated that the longer the treatment period, the more pronounced the accumulation of BUB1B (Figure 7A). Next, we explored whether KIFC1 was involved in the degradation process of BUB1B. The proteasome inhibitor MG132 was added to sh‐KIFC1 and P‐KIFC1 PC cells, and the results demonstrated that KIFC's role in regulating the protein expression level of BUB1B was abrogated (Figure 7B). Moreover, the half‐life of BUB1B was markedly extended in cells exhibiting elevated levels of KIFC1 expression (Figure 7C,D). Our evidence indicates that KIFC1 influences the protein expression level of BUB1B by stabilising the process of BUB1B degradation. To elucidate the precise mechanism through which KIFC1 regulates BUB1B, we conducted ubiquitination experiments. These demonstrated that overexpression and knockdown of KIFC1, respectively, resulted in increased and decreased ubiquitination levels of BUB1B in PC cells transfected with the sh‐KIFC1 and P‐KIFC1 plasmids (Figure 7E). It was previously established that FBXW7 is capable of ubiquitinating BUB1B (Vishnu M. Nair et al.). To further investigate whether KIFC1 competes with FBXW7 to bind BUB1B in PC cells, we designed Co‐IP experiments. The results demonstrated that a reduction in KIFC1 expression resulted in an increase in BUB1B binding to FBXW7 (Figure 7F). In conclusion, the results of these experiments indicate that KIFC1 prevents BUB1B from undergoing ubiquitination‐mediated degradation by competing with FBXW7 for binding to BUB1B.

## 4 Discussion

PC is a highly malignant tumour of the gastrointestinal tract, with an incidence rate almost equal to the mortality rate [33]. Due to its insidious early onset, most cases are already in advanced stages when it is detected. PC can only be eliminated through surgery, but the chances of recurrence after the procedure are high, leading to a poor prognosis [34]. Therefore, finding potential molecular targets for PC may lead to new directions for its treatment. Through a series of experiments, we confirmed that KIFC1 is highly expressed in PC cell lines and patients. KIFC1 overexpression activates the Wnt/β‐catenin pathway through stabilising BUB1B, promoting malignant behaviour and functions in PC cells. These findings indicate that the KIFC1‐BUB1B‐Wnt pathway signalling axis could be a more effective target for PC therapy.

With respect to KIFC1, growing evidence suggests that KIFC1 overexpression is strongly associated with cancer development, progression and drug resistance [35, 36]. Cancer cells typically exhibit centrosome amplification, and these cells survive and proliferate by clustering supernumerary centrosomes for bipolar division [37, 38]. Coincidentally, KIFC1 can aggregate centrosomes to achieve bipolar division of cancer cells, promoting their survival and proliferation [39, 40]. These findings demonstrate that KIFC1 may promote PC progression by facilitating centrosome aggregation in PC cells.

Furthermore, there is evidence that KIFC1 promotes endometrial cancer centrosome amplification by regulating ubiquitination of PLK [41]. FBXW7 plays a key role in inhibiting centrosome replication [42]. Our experiments further explain that in PC cells, KIFC1 relies on a competitive relationship with FBXW7, and this competitive relationship may lead to the retention of amplified centrosomes.

Previous studies have demonstrated that the Wnt/β‐catenin signalling pathway can promote PC tumourigenesis and drug resistance [43, 44]. Targeting this pathway has become a crucial initiative for treating PC and other gastrointestinal tumours [45]. In head and neck carcinoma, KIFC1 has been identified as an'activato' of the Wnt/β‐catenin signalling pathway, promoting the development of head and neck squamous cell carcinoma [46]. Our experimental results suggest that KIFC1 may promote PC development by activating the Wnt/β‐catenin signalling pathway. Further mechanistic exploration revealed that KIFC1 binds to BUB1B and stabilises it at the protein level. Knocking down BUB1B reversed the increase in cell function and Wnt/β‐catenin pathway activity caused by KIFC1 overexpression, suggesting that KIFC1 may activate the Wnt/β‐catenin pathway via BUB1B. BUB1B's function is to inhibit the activity of the anaphase‐promoting complex/cyclosome (APC/C) by blocking the binding of CDC20 to APC/C [47]. BUB1B is a crucial component in chromosome segregation, and BUB1B overexpression induces Aurora‐B hyperactivation, resulting in chromosomal missegregation and aneuploidy [48, 49]. In general, cancer cells with aneuploidy‐containing and dispersed centromeres tend to undergo multipolarisation, preventing them from dividing and leading to cell death [50]. However, KIFC1 promotes the aggregation of dispersed centrosomes in cancer cells, facilitating polar division and ensuring cell survival [39, 40]. The available evidence indicates that KIFC1 has a molecular function to stabilise BUB1B that promotes the proliferation, migration and invasion of PC cells both in vitro and in vivo by activating the downstream Wnt/β‐catenin pathway. In future studies, the precise binding sites and structural basis of the KIFC1–BUB1B interaction will be investigated, with a view to further elucidating the molecular mechanisms underlying KIFC1‐mediated BUB1B stabilisation.

## 5 Conclusion

**FIGURE 8:**
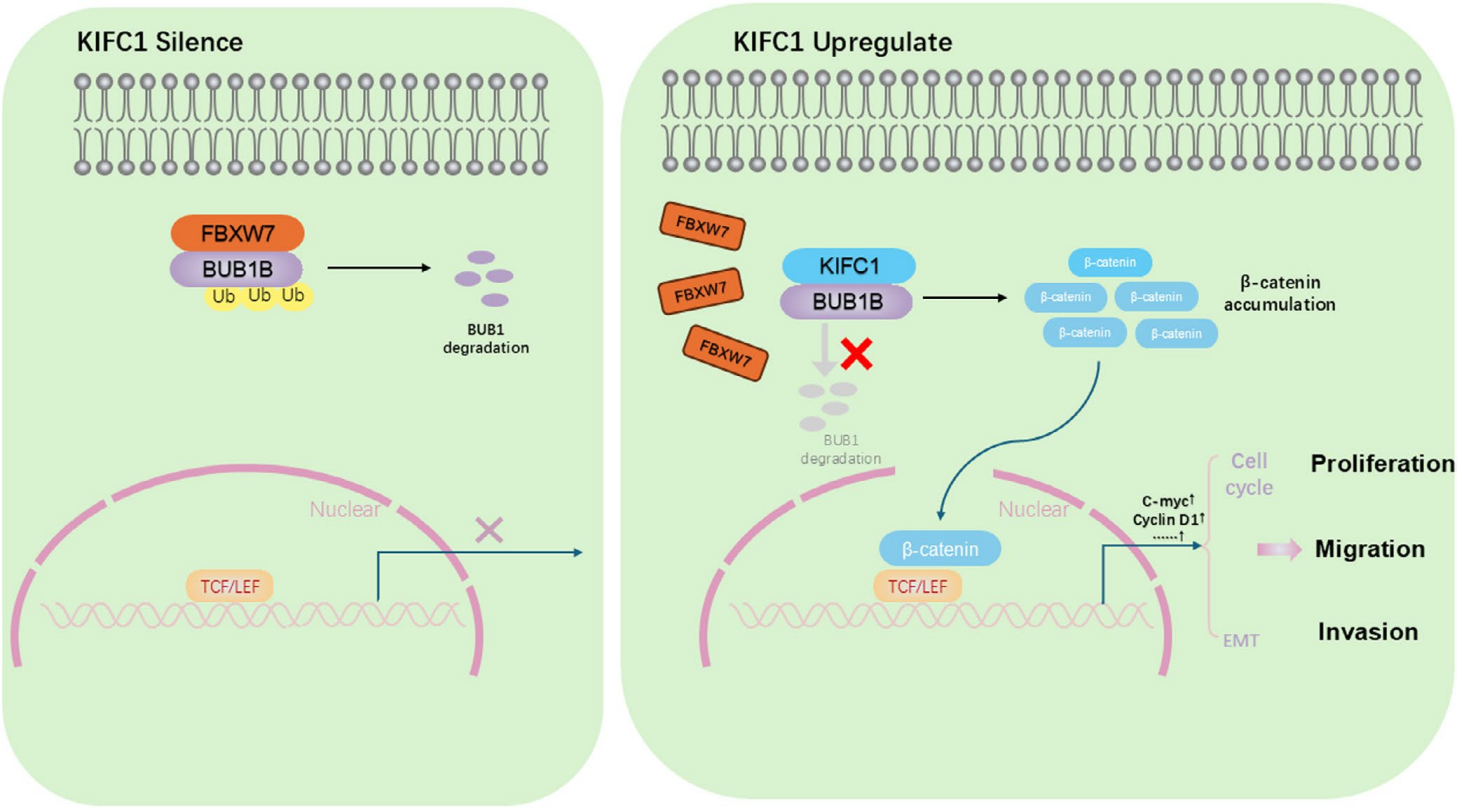
Schematic illustration of the potential molecular mechanism of KIFC1 as a key regulator in PC progression. KIFC1 overexpression promotes PC progression via the BUB1B/WNT/β‐catenin pathway.

In summary, in the clinic, KIFC1 is expressed at high levels in PC tissues and cells, which significantly reduces the overall survival and results in a poor prognosis for patients. Mechanistically, we uncovered that KIFC1 stabilises BUB1B by competitively binding to FBXW7, thereby suppressing BUB1B ubiquitination and subsequent degradation. This stabilisation activates the Wnt/β‐catenin pathway, ultimately driving pancreatic carcinogenesis (Figure 8).

## Author Contributions

**Ao Cui:** data curation (equal), formal analysis (equal), methodology (equal), validation (equal), visualization (equal), writing – original draft (equal). **Ying‐Xue Yu:** resources (equal), validation (equal), writing – original draft (equal). **Mei‐Xue Xiong:** investigation (equal), methodology (equal), validation (equal). **Ji‐Yang Wang:** data curation (supporting), resources (supporting), validation (supporting). **Ye‐Qing Zou:** funding acquisition (supporting), resources (supporting), writing – review and editing (supporting). **Ya‐Qiong Zhu:** funding acquisition (supporting), resources (supporting), writing – review and editing (supporting). **Long‐Jian Ran:** data curation (supporting), resources (supporting), validation (supporting). **Yu Zhang:** data curation (supporting), resources (supporting), validation (supporting). **Rui‐Xiang Liu:** data curation (supporting), resources (supporting), validation (supporting). **Ming‐Yi Dong:** data curation (supporting), resources (supporting), validation (supporting). **Hui Wang:** data curation (supporting), resources (supporting), validation (supporting). **Lu Fang:** funding acquisition (lead), resources (lead), supervision (lead), validation (lead), writing – review and editing (lead). **Xiao‐Wei Fu:** funding acquisition (lead), resources (lead), supervision (lead), writing – review and editing (lead).

## Ethics Statement

The use of human tissue specimens for this study was approved by the Ethics Committee of the Second Affiliated Hospital of Nanchang University (Approval No. Review [2021] No. 039). Animal ethics were reviewed and approved by the Institutional Animal Care and Use Committee of Nanchang Royo Biotech Co. Ltd. (IACUC Issue No: RYE2024033001).

## Conflicts of Interest

The authors declare no conflicts of interest.

## Supporting information


**Data S1.** jcmm70767‐sup‐0001‐FigureS1.pdf.

## Data Availability

Data will be made available on request by contacting the corresponding author.
