# Supplementary material for: KIFC1 Overexpression Promotes Pancreatic Carcinoma Progression via Stabilising BUB1B
Source: J Cell Mol Med. 2025 Aug 26;29(16):e70767. doi: 10.1111/jcmm.70767 (PMC12379544; doi:10.1111/jcmm.70767)

Figure 1G

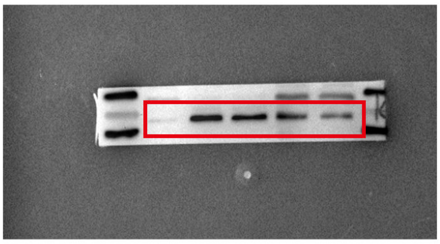

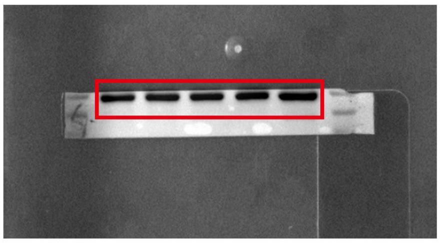

anti-KIFC1 anti-GAPDH

Figure 2A

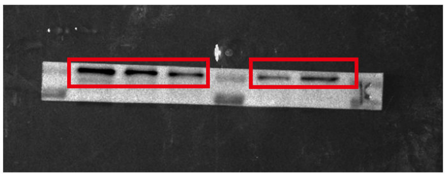

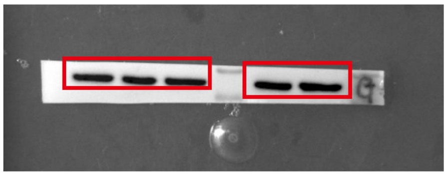

anti-KIFC1 anti-GAPDH

Figure 4C

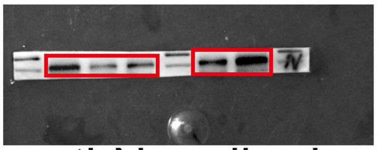

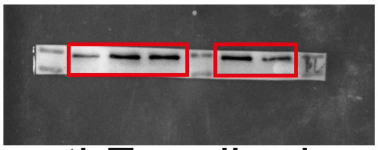

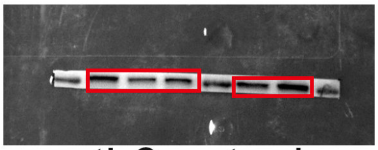

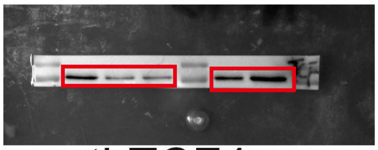

anti-N-cadherin anti-E-cadherin anti-β-catenin anti-TCF4

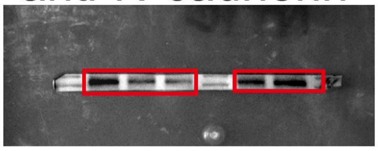

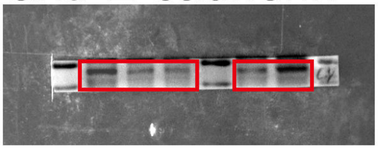

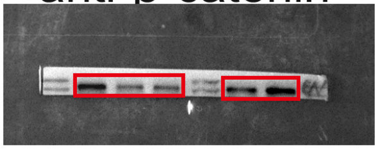

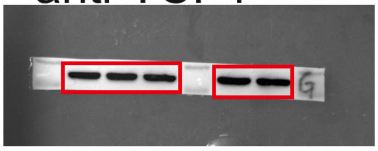

anti-Vimentin anti-c-Myc anti-cyclin D1 anti-GAPDH

Figure 5C

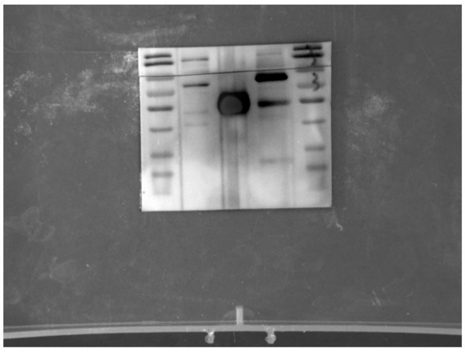

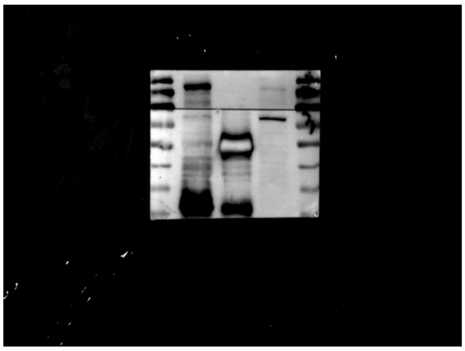

KIFC1 pull BUB1B BUB1B pull KIFC1

Figure 4G

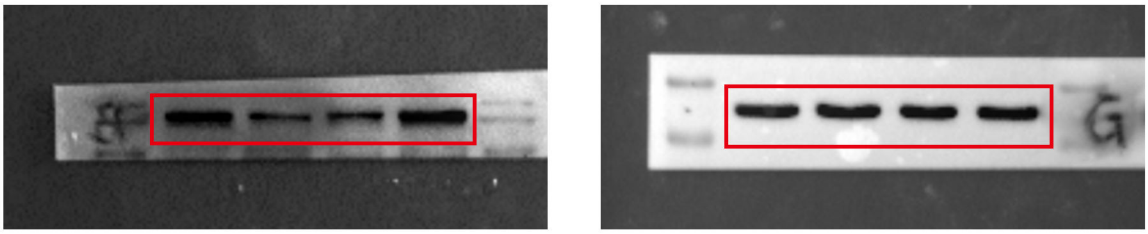

anti-BUB1B anti-GAPDH

Figure 5A

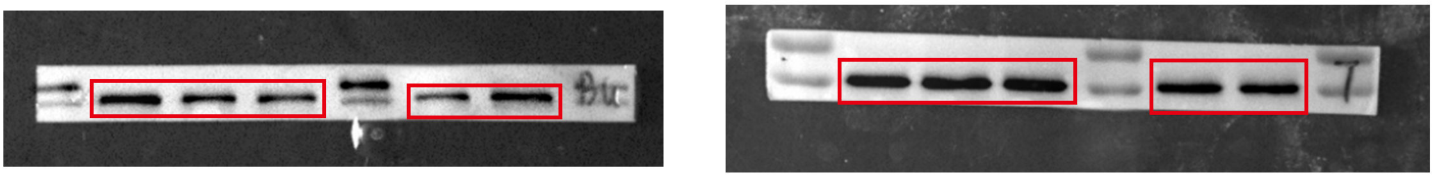

anti-BUB1B anti-GAPDH

Figure 5E

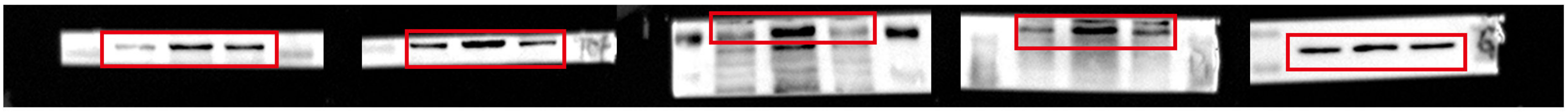

anti-β-catenin anti-TCF4 anti-c-Myc anti-cyclinD1 anti-GAPDH

Figure 6E

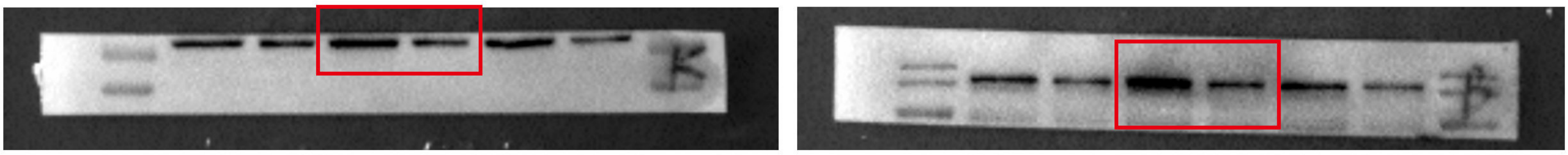

anti-KIFC1 anti-BUB1B

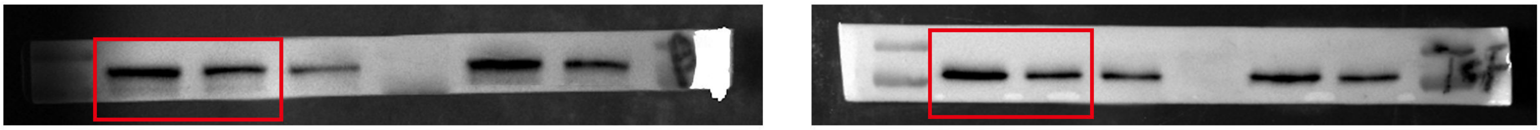

anti-β-catenin anti-TCF4

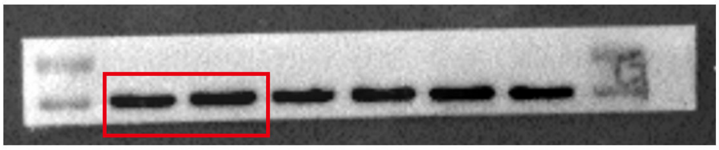

anti-GAPDH

Figure 7A

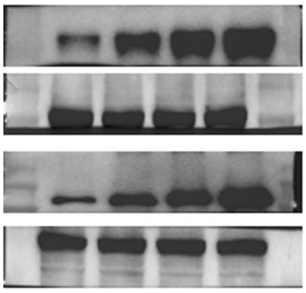

Figure 7B

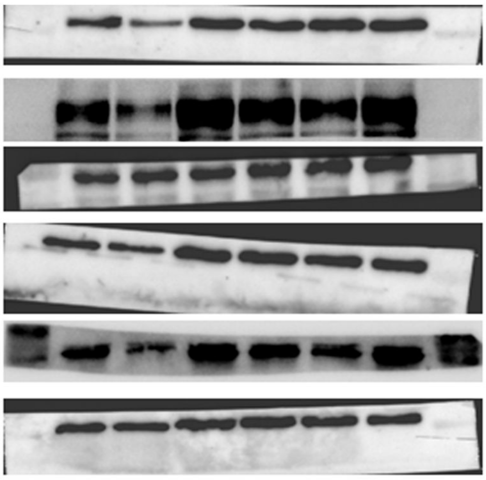

Figure 7C

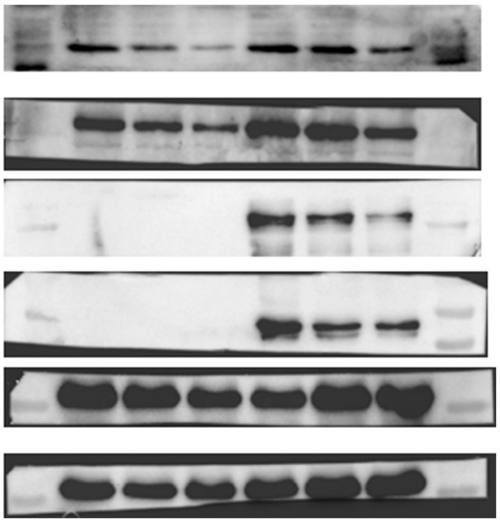

Figure 7E

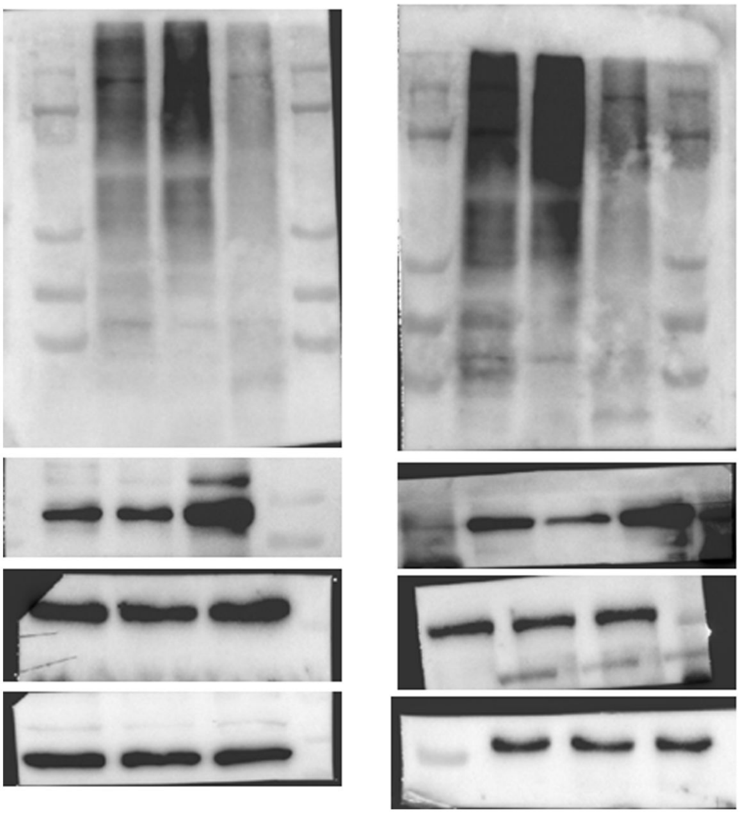

Figure 7F

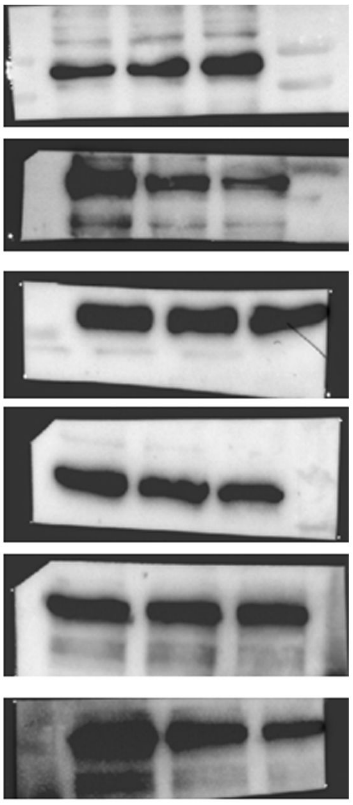

Supplement: Supplementary file 1 — Data S1. jcmm70767‐sup‐0001‐FigureS1.pdf. [file JCMM-29-e70767-s001.pdf]
